# Maternal Antibody Interference and Hybrid Immune Responses to COVID-19 Vaccination in Young Children

**DOI:** 10.21203/rs.3.rs-10581623/v1

**Published:** 2026-08-12

**Authors:** Srividhya Swaminathan, Nadia Bourdoud, Danny Hoi Tsun Chu, Suchaya Osatis, Karissa Choi, Caryl L. Gay, Lakshmi Warrier, Lin Li, Corina Mong, Megan A. Chidboy, Christine Lin, Amayrani Morales, Christine Blauvelt, Gillian Q. Rush, Sirirak Buarpung, Nida Ozarslan, Amelia N. Deitchman, Stephanie L. Gaw, Mary K. Prahl

**Affiliations:** 1. Department of Pediatrics, Division of Pediatric Infectious Disease and Global Health, University of California, San Francisco, CA, United States; 2. Department of Clinical Pharmacy, University of California, San Francisco, CA, United States; 3. Department of Family Health Care Nursing, University of California, San Francisco, San Francisco, CA, United States; 4. Department of Experimental Medicine, University of California, San Francisco, San Francisco, CA, United States; 5. Division of Maternal-Fetal Medicine, Department of Obstetrics, Gynecology, and Reproductive Sciences, University of California San Francisco, San Francisco, CA, United States; 6. Center for Reproductive Sciences, Department of Obstetrics, Gynecology, and Reproductive Sciences, University of California San Francisco, San Francisco, CA, United States

## Abstract

Passively transferred SARS-CoV-2 maternal antibody (MatAb) provides significant protection against COVID-19 hospitalization in infancy, however the impact of MatAb presence and prior COVID-19 infection on the immunogenicity and longitudinal durability of SARS-CoV-2 mRNA vaccination in young children, is not known. Here, we evaluate pre- and post-vaccination and longitudinal SARS-CoV-2 antibody responses of 66 young children aged six months to four years of age. We found that young children mount a robust immune response to mRNA SARS-CoV-2 vaccines regardless of prior infection history. In contrast, MatAb presence at the time of vaccination significantly inhibited the induction and long-term durability of vaccine-induced humoral immunity. Post-primary vaccine IgG levels were not significantly different by biological sex or vaccine manufacturer. MatAb interference resolved by twelve months of age; strategic booster dosing or optimized primary series timing may overcome maternal antibody inhibition and optimize immunologic protection in young children.

## Introduction

Children typically experience milder SARS-CoV-2 disease than adults^1^, but remain at risk for acute COVID-19, particularly young infants^2–4^. The delayed rollout of SARS-CoV-2 vaccination for younger age groups meant many children were infected with the virus before vaccination^3,5^. COVID-19 vaccination studies in infants and children have shown non-inferior neutralizing antibody responses compared to young adults, but little is known about how prior infection influences protective and durable vaccine responses^6,7^. While adult studies of hybrid immunity have revealed distinct immune memory profiles and stronger neutralizing antibody responses^8–11^, data specific to young children is limited^12,13^.

Passively transferred SARS-CoV-2 maternal antibody (MatAbs) provides significant protection against COVID-19 hospitalization in infancy^14,15^, a period of heightened vulnerability to severe disease^2^. SARS-CoV-2 MatAbs persist in infants for several months, with half of infants still with detectable SARS-CoV-2 MatAb at six months of age^14,16–19^. However, MatAbs have been shown to inhibit infant vaccine responses across multiple other immunization platforms^20–25^, potentially through mechanisms such as epitope masking which has been observed in adults receiving SARS-CoV-2 monoclonal antibody therapy^25,26^. It is unknown whether MatAbs similarly interfere with SARS-CoV-2 vaccine-induced immunity in infants.

This study aimed to evaluate how pre-existing immunity, from passively transferred MatAbs or prior COVID-19 infection affects SARS-CoV-2 vaccine-specific antibody responses in infants and young children. These data will help to determine optimal timing and dosing strategies for SARS-CoV-2 vaccines in this vulnerable age group.

## Methods

### Cohort Design and Clinical Data Collection:

The COVIPAL Pediatric Vaccine Study is a prospective cohort study of children, aged 6 months to 4 years of age, that received mRNA SARS-CoV-2 vaccination by their primary care provider. Children were recruited from June 2022 to July 2025, and 66 child participants had at least one blood sample available for analysis (Fig.1A). REDCap surveys were administered to collect information on maternal and child history of vaccination, prior COVID-19 infection, and demographics. Immunization and medical history were confirmed through medical record review.

### COVID-19 Infection and Maternal Antibody Exposure Status

COVID-19 infection and maternal antibody status were defined into four major exposure groups: a COVID-19 naïve group without prior infection with no maternal antibody (COVID-19 naïve MatAb(−)), a hybrid immunity group of young children with COVID-19 infection prior to primary vaccine series follow-up and no maternal antibody (Hybrid Immunity MatAb(−)), COVID-19 naive infants with the presence of maternal antibodies at the time of vaccination (COVID-19 naïve MatAb(+)) and infants with a history of prior COVID-19 infection and the presence of maternal antibody at the time of vaccination (Hybrid Immunity MatAb(+)). Prior COVID-19 infection history was determined by 1) parental reported prior COVID-19 testing by REDCap survey 2) review of the medical record and 3) anti-nucleocapsid antibody testing at each timepoint prior to primary vaccine series follow-up. Participants were categorized as COVID-19 infection naïve if they had no parental reported prior COVID-19 positive testing by REDCap survey and no medical record history of positive COVID-19 testing and negative anti-nucleocapsid antibody testing at any timepoint prior to the primary vaccine series follow-up timepoint. Subsequent COVID-19 infection after the primary vaccine series timepoint during longitudinal follow-up was not considered in the initial categorization. Participants were categorized as “Hybrid immunity” if they had any positivity in the previously mentioned testing. Infants were considered maternal antibody positive (“COVID-naïve MatAb(+)”) if they were RBD IgG positive at the pre-vaccine time point and ≤ 12 months of age and had a negative clinical history for COVID-19 infection and had negative nucleocapsid antibodies prior to primary series follow-up time point. Infants with either a history of COVID-19 infection during the time when passive maternal antibody would be present (<7 months of age) or positive anti-nucleocapsid testing at the pre-vaccine timepoint (in case of asymptomatic infection) and age <12 months were categorized as “Hybrid immunity MatAb(+)”. Additional comparison groups included historic pregnancy/lactation and healthy adult female biospecimen vaccination cohorts as previously detailed in^27,28^. The Institutional Review Board of University of California San Francisco approved these studies (IRBs 20–32077 and 19–29713); written informed consent was obtained from each participant.

### Sample collection and plasma isolation

Blood was obtained by either venipuncture or heel stick blood draw, collected in EDTA-coated tubes. Plasma was separated by centrifugation and immediately cryopreserved until analysis.

### Measurement of SARS-CoV-2 specific antibody

IgG antibody against Receptor- Binding Domain (RBD), Nucleocapsid (N), Spike subunit (S1 and S2) was analyzed using 4 -plex panel kit (Bio-Rad, 12014634). IgA and IgM antibodies against the spike receptor RBD (Bio-Rad, 12015406) or Nucleocapsid (N) (Bio-Rad, 12014773) of SARS-CoV-2 were analyzed with a multiplex-based human serology kit (Bio-Rad, 12014777) according to the manufacturer’s protocol as we have previously reported^27,28^. Infant plasma samples were diluted in the ratio of 1:1000 for IgG detection, and 1:100 for IgA and IgM detection. To minimize potential IgG interference during IgM quantification, serum samples were pre-treated with GullSORB IgG inactivation reagent (Meridian Bioscience) at a 1:10 ratio before further dilution. Diluted plasma samples were incubated with 4-plex or RBD or N protein–conjugated beads for 30 minutes at room temperature. Respective secondary antibodies for IgG, IgM, and IgA were added to the wells and incubated at room temperature for 30 minutes. This was followed by a 10-minute incubation with streptavidin-phycoerythrin (SAPE) to label the complexes. After a final wash and bead resuspension, the plates were analyzed using a Bio-Plex 200 system (Bio-Rad). Results were expressed as median fluorescence intensity (MFI) per 100 beads. Cutoffs were determined using a similar criterion as previously reported^14,27,28^. Positive cutoffs for MFI were 75 and 100 for IgG anti-N and anti-RBD, 80 and 100 for IgA anti-N and anti-RBD, and 250 and 600 for IgM anti-N and anti-RBD.

### Statistical analysis:

All analyses were performed using GraphPad Prism (version 10), Python (version 3.14), and/or R (version 4.4.6). Descriptive analysis included medians, means, interquartile ranges (IQRs), standard deviations, and ranges for continuous variables. Mann-Whitney U test was used for two-group comparisons. Associations between continuous variables were assessed by Spearman’s rank correlation (R_s_) including pre- and post- vaccine IgG levels. Where appropriate, regression lines and splines were included in graphs to visualize trends in data. Two-sided p-values were calculated for all test statistics, and p<0.05 was considered significant. Quantification of longitudinal IgG responses were analyzed using segmented mixed-effects models^29^. A participant-level heatmap visualized the breadth of IgG responses across N, RBD, S1, and S2 antigens.

## Results

### Child Participant Characteristics

A total of 66 infants were included in our study of which 50% had maternal antibody at the time of SARS-CoV-2 vaccination and 39.4% had COVID-19 infection prior to the primary vaccine series follow-up timepoint, additional participant characteristics are listed in Table 1.

### Maternal antibody presence restricts infant SARS-CoV-2 vaccine IgG responses

We first explored the relationship between infant age and levels of MatAb at the pre-vaccine time point. Consistent with our prior study^28^, we found the persistence of maternally derived SARS-CoV-2 IgG up to 12 months of age, with decreasing levels of IgG with increasing age (R_s_=−0.3949, p=0.0279, Fig.1B).

We categorized our pediatric cohort by prior COVID-19 infection and MatAb status and compared post-primary vaccine series SARS-CoV-2 IgG levels to RBD, S1, and S2 antigens across exposure groups.

We first found that COVID-19 naïve MatAb(+) infants had significantly restricted absolute post-primary vaccine RBD IgG response (median=1,754, IQR [512.5–4,264], MFI), in comparison to children in which MatAb had cleared prior to vaccination (MatAb(−)), in both naïve and hybrid immunity subgroups (p<0.0001 and p<0.0001, respectively Fig.1C). An additional group of infants previously COVID-19 infected at an age when they would still have the presence of maternal antibody (Hybrid immunity MatAb(+), age range at time of COVID-19 infection 3–7 months), displayed a high variability of post-primary vaccine RBD IgG responses (median=4,616, IQR [990.7–12,451]), which was lower than the COVID-19 naïve MatAb(−) group (p=0.0245), but not different than other exposure groups.

Since infants with maternal antibody have the presence of the passively derived antibody at the time of vaccination but are not T or B cell primed to SARS-CoV-2 antigen, we next explored the post-primary vaccine series IgG fold-change (Fig.1D). Strikingly, the COVID-19 naïve MatAb(+) cohort demonstrated a minimal RBD IgG fold change (median=2.051, IQR [0.583–6.364] MFI) which was significantly lower than the COVID-19 naïve MatAb(−) and the hybrid immunity MatAb(−) groups (p<0.0001 and p=0.0004 respectively). Additionally, the fold-change RBD IgG of hybrid immunity MatAb(+) infants was lower than COVID-19 naïve MatAb(−) children (p<0.0001), but not different than the hybrid immunity MatAb(−) group.

We explored if MatAb related post-primary vaccine IgG inhibition was maternal antibody level dependent and found that in our COVID-19 naïve MatAb(+) infants’ pre-vaccine RBD IgG levels were not correlated with the post-vaccine RBD IgG levels (Rs=−0.2211, p=0.3490 (Fig.1E). Consistent with the RBD IgG data, post-vaccine absolute S1 IgG levels and fold-change were significantly lower in COVID-19 naive MatAb(+) cohort when compared to both COVID-19 MatAb(−) (p<0.0001 and p<0.0001, respectively) and the hybrid immunity MatAb(−) groups (p<0.0001 and p=0.0042, **Supplemental Fig.1A,B).** Analysis of S2 subunit revealed a similar but distinct pattern, with post-vaccine COVID-19 naïve MatAb(+) absolute S2 levels lower than the hybrid immune MatAb(−) (p<0.0001, **Supplemental Fig. 1C**), and interestingly both the COVID-19 naïve MatAb(+) and hybrid immunity MatAb(+) groups mounted a substantial increase in S2 fold-change post-vaccination overall, but were lower compared to the COVID-19 naive MatAb(−) group (p=0.0011 and p=0.0011, respectively, **Supplemental Fig. 1D).** Similarly to RBD in our COVID-19 naïve MatAb(+) group, no correlation was found between pre-vaccine (MatAb) to post-vaccine IgG levels of S1, but there was a significant inverse correlation pattern with S2 pre-vaccine to post-vaccine IgG levels (Rs=−0.4617, p=0.0405 **Supplemental Fig. 2A,B).**

Taken together, this indicates that maternal antibody significantly restricts the post-vaccination SARS-CoV-2 IgG response in infants, but it is not strictly dependent on absolute levels of maternal IgG.

### Robust SARS-CoV-2 vaccine mediated immunogenicity in young children with and without prior COVID-19 infection

We next compared the absolute magnitude of RBD-specific IgG antibody levels across the MatAb(−) exposure groups. Both the COVID-19 naïve MatAb(−) and the hybrid immunity MatAb(−) group mounted an overall robust RBD IgG response to primary SARS-CoV-2 vaccination (median=10,983, IQR [8,073–12,300], and median=13,301, IQR [10,126–15,896] MFI respectively, Fig.1C), and the absolute levels were not different between groups. We next analyzed vaccine responses to SARS-CoV-2 Spike subunits S1 and S2. In contrast to RBD, post-primary absolute S1 and S2 IgG levels were higher in the hybrid immunity MatAb(−) group as compared the COVID-19 naïve MatAb(−) group (p=0.0469 and p=0.0009 respectively **Supplemental Fig.1A,C**).

We next analyzed the fold-change of RBD IgG post-vaccine levels over pre-vaccine baseline RBD IgG levels among groups (Fig.1D). The COVID-19 naïve MatAb(−) group displayed the highest fold-change of RBD IgG (median=3,203, IQR [2,204–6,441] MFI) likely representing the absence of baseline immunity. Moreover, young children with hybrid immunity had a robust fold-change RBD IgG increase over baseline to vaccination (median=42.46, IQR [11.99–2,125] MFI), but this was significantly lower than the fold-change of the COVID-19 naïve group (p=0.0008). Similarly, fold-change comparisons followed the same pattern with S1 fold-change lower in the hybrid immunity MatAb(−) group compared to the COVID-19 naïve MatAb(−) group (p=0.0002). The S2 fold change comparisons across exposure groups were not significantly different (**Supplemental Fig.1B,D**).

Together, this shows that young children, both COVID-19 naïve children and children with hybrid immunity, had a robust serologic response to SARS-CoV-2 mRNA vaccination.

### Longitudinal durability of SARS-CoV-2 IgG responses

We next analyzed the durability of SARS-CoV-2 IgG responses over two-year follow up, including interim booster doses and COVID-19 infections during follow-up timepoints (Fig.2A). Overall, we found that the MatAb(−) groups maintained a high level of sustained serologic RBD IgG response >3500 MFI, with waning prior to and robust responses following booster doses. In contrast, the COVID naïve MatAb(+) group had lower levels of RBD IgG over the first year of follow-up and did not reach levels of ~3000 MFI until after 2023–2024 booster dose. Similar, patterns were observed with longitudinal anti-S1 levels, though anti-S2 IgG levels were overall lower magnitude in all exposure groups **(Supplemental Fig.3A,C**).

To quantify the impact of MatAb(+) on longitudinal durability of RBD IgG to SARS-CoV-2 vaccination, we employed segmented linear mixed effect models to characterize apparent biphasic production and decay. We found that the presence of MatAb not only inhibits the initial induction and boosting responses to primary vaccination (157.6 MatAb(−) vs 116.9 MatAb(+) anti-RBD IgG units/day, p<0.001), but it also restricts reduces the longitudinal durability of SARS-CoV-2 IgG responses (5.39 MatAb(−) vs −35.3 MatAb(+) anti-RBD IgG units/day, p<0.001) (Fig.2B). Similar trends were observed for anti-S1 and anti-S2 responses (**Supplemental Fig.3B,D**, **Supplemental Table 1**).

### Age dependent maturation of humoral vaccination responses

To further pinpoint age-dependent vaccine-responsiveness, we evaluated post-primary RBD IgG level by children’s age in months. We found a positive correlation between post-primary RBD IgG level and child’s age (R_s_=0.5244, p<0.0001). Moreover, there was a sharp inflection point around 12 months of age when MatAb had waned (Fig. 3A). Moreover, we found that age-related grouping revealed suppression of antibody levels in the first year of life. Infants between 6–12 months of age demonstrated the lowest post-primary vaccine IgG level compared to a strong recovery of immunogenicity in 12–18 month olds with threefold increase in antibody levels (p<0.0001). Beyond 18 months, the anti-RBD IgG levels decreased, but stabilized across the age groups (Fig.3B). Similar to RBD profile, post-primary anti-S1 and anti-S2 IgG levels demonstrated inflection point at 12 months of age **(Supplemental Fig. 4A,B)** and increased in immunogenicity between 12–18 months **(Supplemental Fig. 4C,D).**

To compare antibody responses across the age spectrum, we compared both our COVID-19 naïve children (MatAb(−) and MatAb(+)) to two historical COVID-19 naive vaccine adult cohorts after primary vaccine series 1) pregnant/lactating mothers and 2) healthy (non-pregnant non-lactating) female adults. We found that children had similar post-vaccination SARS-CoV-2 IgG levels to both pregnant/lactating mothers, and healthy adult females. However, MatAb(+) infants remained lower than both COVID-19 naïve children (p<0.0001), as well as compared to pregnant/lactating mothers and health adult females (p<0.0001 and p<0.0001 respectively, Fig. 3C).

Together, this data suggests that early life post-primary vaccine restriction resolves after one year of age.

### Vaccine immunogenicity is independent of biological sex and vaccine manufacturer.

To assess the impact of biologic sex on vaccine responses, we compared post-primary anti-RBD, S1, and S2 IgG responses between male and female children and found no statistically significant difference across exposure groups (Fig.4A, **Supplemental Fig. 5A,B**). Next, we compared the post-primary anti-RBD IgG levels between participants receiving the 2-dose Moderna (Spikevax) and the 3-dose Pfizer (Comirnaty) and stratified by MatAb status (Fig. 4B). Within both the MatAb(+) and MatAb(−) cohort, RBD, S1, and S2 antibody responses were not statistically different between vaccine manufacturers (Fig. 4B, **Supplemental Fig. 5C,D**). This indicates that the modulation of vaccine responses is independent of biologic sex and vaccine manufacturer.

### Rapid waning of SARS-CoV-2 infection induced nucleocapsid antibody levels

To understand the durability of infection-induced antibodies, we evaluated the longitudinal kinetics of anti-nucleocapsid (N) IgG levels by months since confirmed COVID-19 infection (Fig. 4C). Anti-N IgG are generated after exposure to whole virus and is commonly used to indicate exposure to natural infection. Most participants exhibited high levels of initial IgG levels (range 93.5 to 6337.5 MFI) in the first 6 months postinfection, but anti-N IgG levels rapidly decayed over time; most reverted to seronegative between 6–12 months post-infection.

Lastly, participant level heatmap across SARS-CoV-2 antigens at pre- and post-primary vaccine timepoints visualized the breadth of antibody responses across the cohort (Fig. 4D).

### Longitudinal kinetics of vaccine induced IgA and IgM antibodies

To understand vaccine responses beyond the IgG isotype, we evaluated longitudinal trajectories of plasma anti-RBD and anti-N IgA and IgM antibodies throughout the primary series and subsequent booster doses in a subset of participants **(n=50, Supplemental Fig. 6)**. Overall, anti-RBD and anti-N IgA responses **(Supplemental Fig. 6A,B)** were modest with only n=4 and n=5 respectively with any positivity over the course of the study. Similarly, only 3 participants had positive anti-RBD IgM responses **(Supplemental Fig. 6C,D)** during any longitudinal timepoint. Overall, plasma IgA and IgM longitudinal responses revealed minimal positivity and classic kinetic profiles with rapidly waning.

## Discussion

Our study of revealed that young children have robust induction of anti-RBD IgG after COVID-19 mRNA vaccination, in both naïve and infants with prior COVID-19 exposure. Notably, we observed a striking blunting of infant vaccine-induced antibody responses when maternal antibody (MatAb) was present at time of vaccination; this inhibition resolves by one year of age.

Our study demonstrates that young children mount a robust immune response to mRNA SARS-CoV-2 vaccines, but MatAb present at the time of vaccination significantly inhibit both the initial response and the long-term durability of vaccine-induced immunity. To our knowledge, this study is the first to show that passive maternal SARS-CoV-2 IgG profoundly blunts infants’ active immune responses following mRNA vaccination. While MatAb interference is documented across other traditional vaccine platforms^21–25^ — and has led to a global recommendation of delaying measles vaccination until maternal antibodies wane between 9 to 12 months^24,30^— the exact mechanism remains undefined. Potential proposed mechanisms for this inhibition have included epitope masking— as recent studies including a mouse model using plasma from mRNA-vaccinated adults and a study of adults receiving SARS-CoV-2 monoclonal antibodies have shown altered post-vaccination antibody and memory B cell responses^26,31^. Our findings suggest that MatAb interference was not strictly maternal antibody level-dependent, contrasting with prior research on other vaccines^21,22,24,25^. Future studies focusing on epitope masking could provide critical insights into these mechanisms and inform vaccine design including tailored formulations for prenatal versus infant vaccination administration.

Our data also identifies a key immunological inflection point at 12 months of age. Infants vaccinated between 6 and 12 months exhibited the lowest post-primary series vaccine levels, while those aged 12–18 months showed a threefold recovery in vaccine-mediated immunogenicity. However, maternal antibody interference constrained the longitudinal immune responses in young infants, with lower RBD IgG levels persisting throughout the first year. Interestingly, some children receiving the 2023–2024 monovalent booster recovered antibody levels similar to those seen in older children.

Epidemiological data has shown that infants under 6 months are among one of the highest-risk groups for COVID-19-related hospitalization, particularly during the early Omicron wave^32,33^. However, little is known about hybrid immunity in children under 4 years of age, whose immune profiles differ from older children and adults^34^. While hybrid immunity has been shown to enhance antibody responses in adults and older children^35,36^, our data reveal a more nuanced picture in young children. Post-vaccine RBD IgG absolute levels were similar in COVID-19 naïve and hybrid immunity children; although hybrid-immunity children showed a robust post-vaccination increase, their fold-change was lower than that of naïve children, likely reflecting baseline pre-existing immunity. Hybrid immunity did broaden the breadth of immune responses, yielding higher IgG levels against S1 and S2. This indicates that young children with prior COVID-19 infection still derive immunological benefits from vaccination, as do COVID-19 naïve children.

We found that pediatric vaccine immunogenicity is consistent across biological sex and vaccine manufacturers. Antibody levels were comparable between children receiving higher dosage 2-dose Moderna (Spikevax) or lower dosage 3-dose Pfizer (Comirnaty) series, when controlled for MatAb status. We observed minimal plasma IgA and IgM responses, consistent with our prior study in lactating mothers and breastfeeding infants^28^, indicating that mRNA vaccination primarily drives a systemic IgG response. Additionally, the rapid waning of anti-Nucleocapsid (N) IgG in our cohort highlights the limitations of using N-seropositivity as a marker for prior infection in children, as most reverted to seronegative within 6–12 months.

This study highlights the immunologic benefits of SARS-CoV-2 vaccination in infants and young children under 4 years of age, regardless of prior COVID-19 exposure history. However, the diminished immunogenicity observed in infants vaccinated in the presence of maternal antibodies suggests either that either an additional booster dose at 12–18 months of age or delaying the primary vaccine series until one year of age may be beneficial. Decisions on vaccine schedules should consider evolving epidemiological trends, emerging variants, and infant hospitalization rates.

## Supplementary Material

Supplementary Files

This is a list of supplementary files associated with this preprint. Click to download.

- SupplementaryInformationNatureCommunications.docx

## Figures and Tables

**Figure 1:**
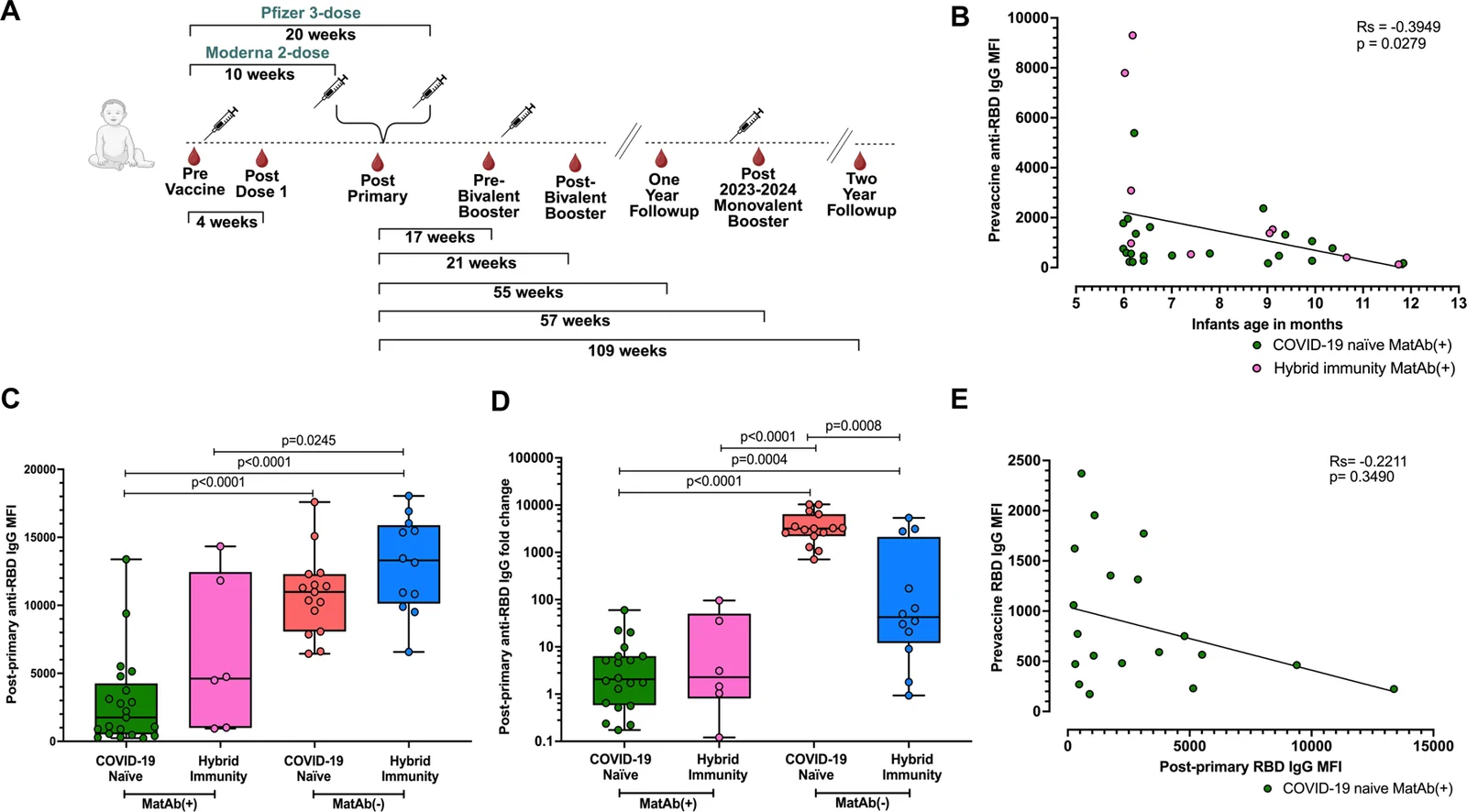
Study timeline and post-primary vaccine series anti-RBD IgG responses by exposure group **A)** Study timeline for SARS-CoV-2 mRNA vaccination schedule and blood collection timepoints for infants and young children receiving either 2-dose Moderna (Spikevax) or 3-dose Pfizer (Comirnaty). Infant/Child blood (red drops) was collected at eight potential time points, and bars indicate the average weeks between timepoints for pediatric participants. Syringe icons indicate vaccine dose administration. Post-primary vaccine series **B)** Pre-vaccine RBD IgG antibody MFI levels correlated with infant age in months restricted to maternal antibody (MatAb(+), n=31) infants to demonstrate passive maternal antibody decay. Circles represent COVID-19 naïve MatAb(+) (green circles) and Hybrid immunity MatAb(+) (pink circles) cohorts. The correlation line represents the linear regression fit for visualization only. Statistical correlation was assessed using Spearman’s rank correlation coefficient (R_s_). **C)** Anti-RBD IgG absolute levels MFI and **D)** Anti-RBD IgG fold-change post-primary vaccine series MFI levels over pre-vaccine MFI levels by exposure group: COVID-19 naïve MatAb(+) (n=21 for C and n=20 for D), Hybrid immunity MatAb(+) (n=6), COVID-19 naïve MatAb(−) (n=15), Hybrid immunity MatAb(−) (n=12). Data presented as box and whisker plots represent median and IQR. Two-sided *p* values were calculated for all test statistics by Mann-Whitney U test, p<0.05 was considered significant, and significant values are indicated in graph brackets. **E)** Correlation between pre-vaccine RBD IgG MFI levels (passive maternal antibody) and post-primary vaccine IgG levels in the COVID-19 naïve MatAb(+) cohort (green circles, n=20). The correlation line represents the linear regression fit for visualization only. Statistical correlation was assessed using Spearman’s rank correlation coefficient (R_s_).

**Figure 2:**
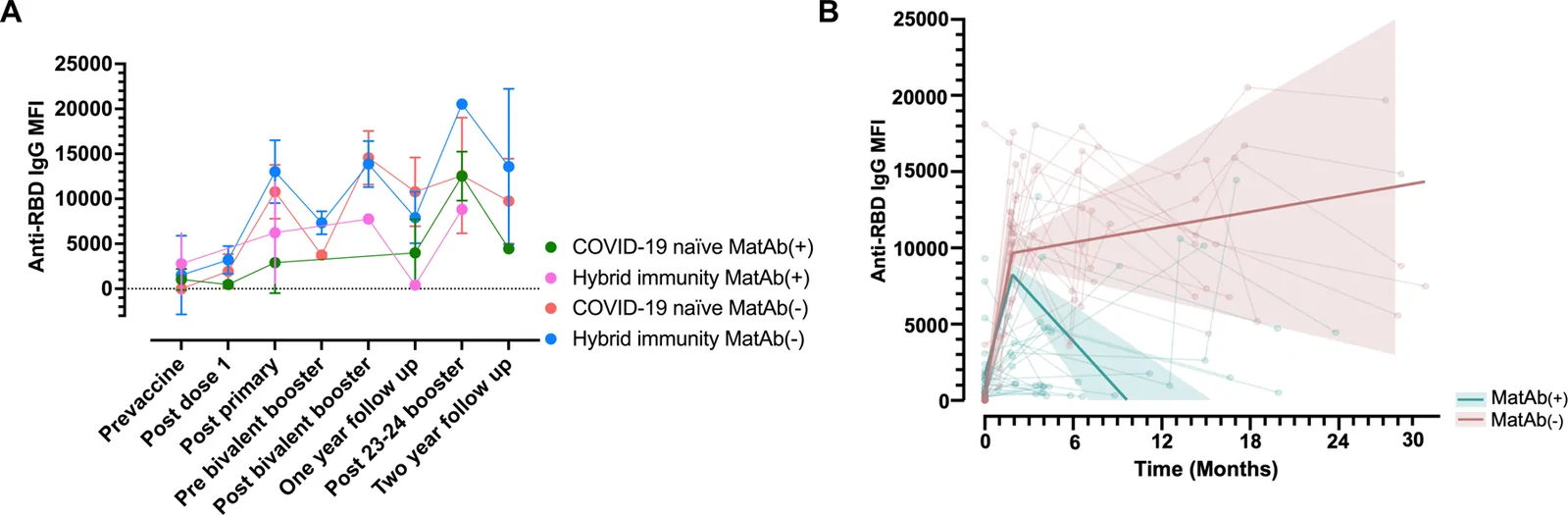
Longitudinal durability of anti-RBD IgG responses by exposure groups **A)** Longitudinal graph of anti-RBD IgG antibody trajectory across timepoints pre-vaccine to two-year follow-up across exposure groups. Each connected data point represents the mean and standard deviation at each time point, stratified by exposure status. Infection status determined prior to primary vaccine follow-up timepoint **B)** Segmented linear regression model fit of anti-RBD level over time by presence or absence of MatAb. Bold lines and shaded area are model prediction and 50% prediction intervals. Connected points represent raw data by participant.

**Figure 3:**
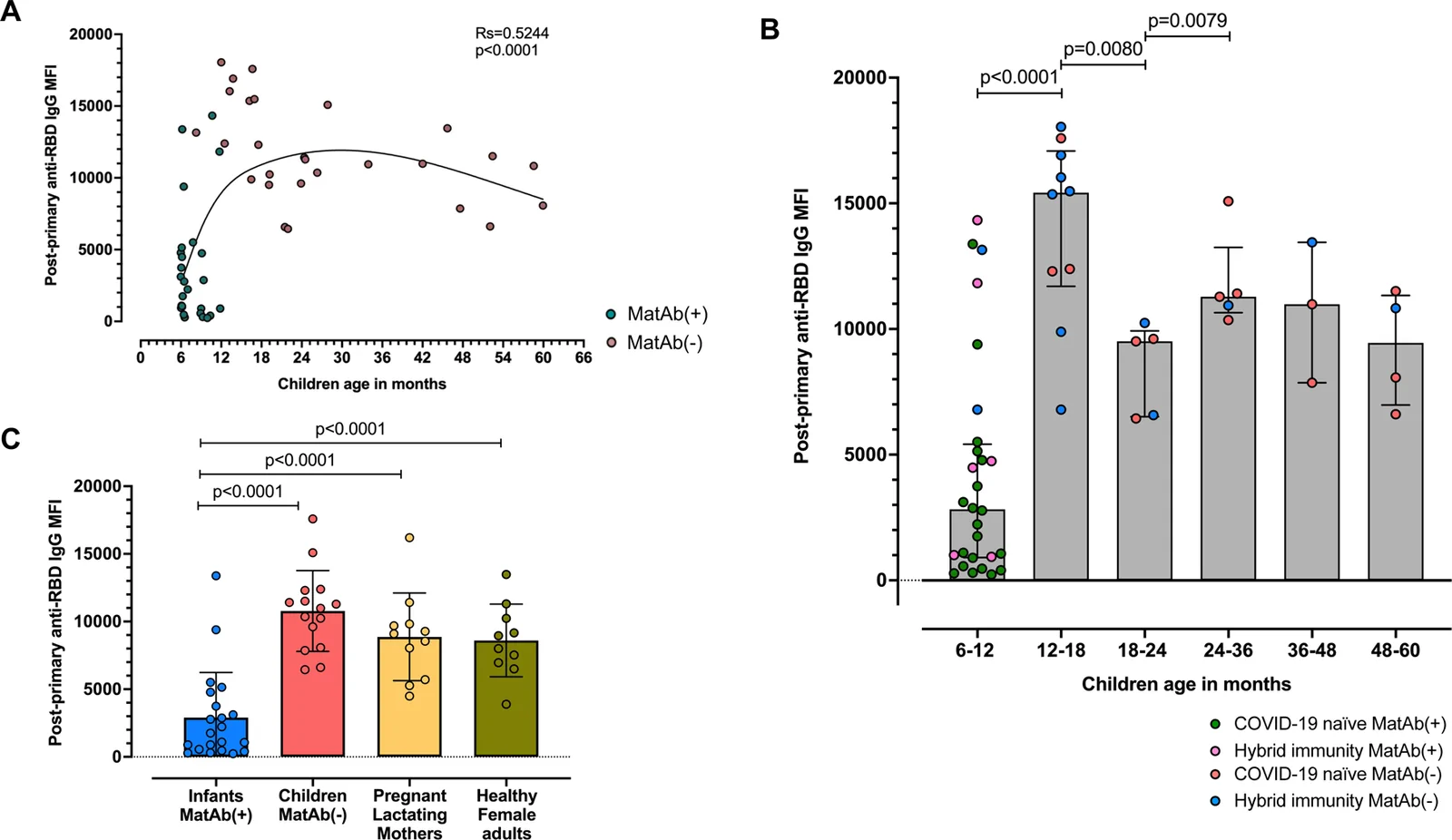
Age-dependent SARS-CoV-2 IgG vaccine-induced immunogenicity **A)** Correlation of post-primary vaccine series RBD IgG by children age in months, with MatAb(+) infants in teal circles (n=27) and MatAb(−) children (n=27) in mauve. Line represents spline for visualization only, statistical correlation by Spearman’s rank correlation coefficient. **B)** Post-primary anti-RBD IgG MFI levels stratified by age groups: 6–12 months (n=28), 12–18 months (n=10), 18–24 months (n=5), 24–36 months (n=5), 36–48 months (n=3), 48–60 months (n=4). Circles color by exposure group. Two-sided *p* values were calculated for all test statistics by Mann-Whitney U test, p<0.05 was considered significant, and significant values are indicated in graph brackets. Bars represent median with IQR. **C)** Post-primary vaccine series anti-RBD IgG levels between COVID-naïve: infants (MatAb(+), n=21), children (MatAb(−), n=15), pregnant/lactating mothers (2-dose series, n=11), and healthy female adults (2-dose series, n=10). Two-sided *p* values were calculated for all test statistics by Mann-Whitney U test, p<0.05 was considered significant, and significant values are indicated in graph brackets. Bars represent median with IQR.

**Figure 4:**
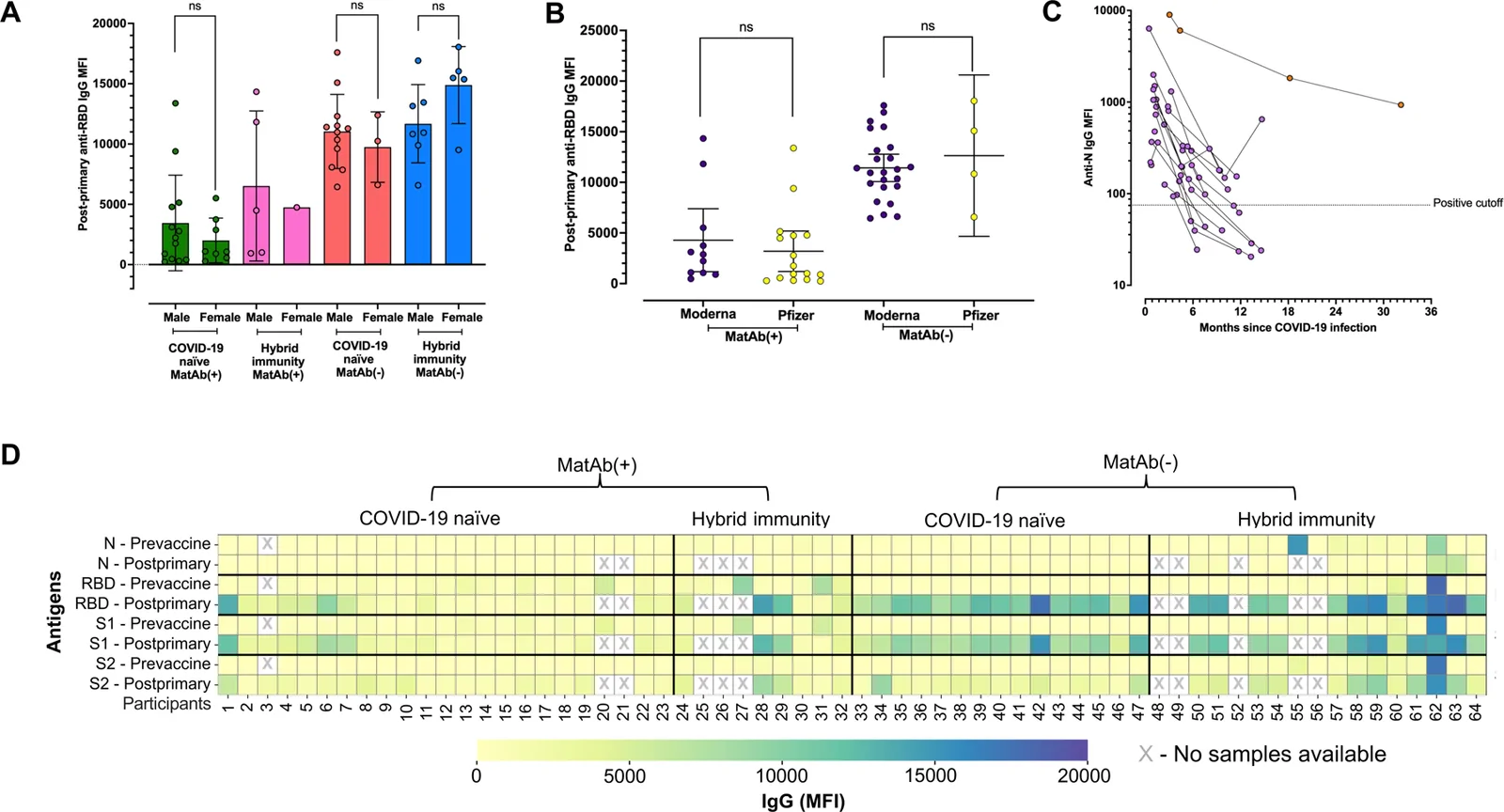
Post-vaccination RBD IgG responses by biological sex and vaccine type, anti-nucleocapsid antibody decay kinetics, and participant heatmap **A)** Post-primary series anti-RBD IgG levels by biological sex stratified by exposure group. Box and whisker plots represent median with IQR with participant data points. Groups include: COVID-19 naïve MatAb(+) (Male (n)= 13, Female (n)= 8) and Hybrid immunity MatAb(+) (Male (n)= 5, Female (n)= 1), COVID-19 naïve MatAb(−) (Male (n)= 12, Female (n)= 3), Hybrid immunity MatAb(−) (Male (n)= 7, Female (n)= 5), **B)** Post-primary series anti-RBD IgG levels by vaccine manufacturer: MatAb(+) infants who received Moderna (n = 11) or Pfizer (n = 16) and MatAb(−) infants who received Moderna (n = 24) or Pfizer (n = 4). Statistical analyses for biological sex and vaccine manufacturer were performed and two-sided *p* values were calculated for all test statistics by Mann-Whitney U test, p<0.05 was considered significant. **C)** Longitudinal decay of anti–nucleocapsid (N) IgG antibody responses are plotted as MFI against time since confirmed COVID-19 infection (in months). The scatter plot represents the pediatric participants (n=31) with a history of a documented COVID-19 infection prior to enrollment (purple), one pediatric participant had two documented COVID-19 infections prior to enrollment and primary vaccination series (orange). The connecting lines track the decline of antibody level within the same subject over time. The horizontal dotted line denotes the seropositivity threshold of 75 MFI for anti-N IgG. **D)** Participant level heatmap of the pediatric participants with either pre-vaccine or post-primary vaccine timepoints available (n=64) the breadth of IgG responses to nucleocapsid (N), receptor-binding domain (RBD), S1, and S2 antigens based on exposure groups. Each cell represents individual antibody levels in MFI with lower values indicated in yellow to higher values in dark teal against SARS-CoV-2 antigens. ‘X’ indicates unavailable sample at timepoint.

**Table 1:** Child Participant Characteristics

Child Characteristic	Infant/Child Participants Total n=66 n (%)
**Presence of maternal antibody at time of 1**^**st**^ **vaccination dose**	
Maternal antibody negative	33 (50%)
Maternal antibody positive	33 (50%)
**COVID-19 infection prior to post-primary series follow-up timepoint**	
COVID-19 naive	40 (60.6%)
Prior COVID-19 infection	26 (39.4%)
**Vaccine Type**	
Moderna (Spikevax)	40 (60.6%)
Pfizer (Comirnaty)	26 (39.4%)
**Sex**	
Female	20 (30.3%)
Male	46 (69.7%)
**Prematurity**	
Full term	62 (93.9%)
Preterm <37 weeks	4 (6.1%)
**History of maternal SARS-CoV-2 vaccination prior to delivery**	
No vaccine prior to delivery	20 (30.3%)
Vaccine prior to delivery	46 (69.7%)
**Self-described race/ethnicity**	
American Indian or Alaska Native	0 (0%)
Asian	12 (18.2%)
Black or African American	0 (0%)
Hispanic/Latina	1 (1.5%)
Native Hawaiian or Other Pacific Islander	0 (0%)
White or Caucasian	39 (59.1%)
More than one race	14 (21.2%)
**Child health conditions prior to enrollment** *	
Congenital Malformation	4 (6.1%)
Lung Condition	1 (1.5%)
Thyroid Condition	1 (1.5%)
Other Condition^#^	3 (4.5%)
None or not reported	57 (86.3%)
	Mean (SD, range)
**Infant age at 1**^**st**^ **vaccine dose**	17.5 (14.9, 6–60)
**Child age in months at 1**^**st**^ **COVID-19 infection**	26.6 (20.0, 3–78)

* Enrollment survey reported no childhood diabetes, asthma, heart condition, blood clots, depression, anxiety, cancer, or immunodeficiency.

# Other parent-reported child health conditions at enrollment included: Oral aversion/g-tube feed, 22q11 microduplication; macrocephaly; truncal hypotonia, Left-sided SVC benign cardiac condition.

## Data Availability

Deidentified study data, along with the data dictionary, will be made available upon reasonable request to the corresponding author for academic research purposes, after publication, under a data-sharing agreement.
